# Investigation of the Immunogenic Properties of Ovalbumin Modified by Urban Airborne Particulate Matter

**DOI:** 10.1007/s00005-023-00679-8

**Published:** 2023-05-28

**Authors:** Bernadeta Nowak, Anna Wądołek, Olga Mazuryk, Anna Poznańska, Katarzyna Majzner, Grzegorz Majka, Maria Oszajca, Małgorzata Barańska, Grażyna Stochel, Janusz Marcinkiewicz

**Affiliations:** 1 Department of Immunology, Faculty of Medicine, Jagiellonian University Medical College, Kraków, Poland; 2 Department of Inorganic Chemistry, Faculty of Chemistry, Jagiellonian University, Kraków, Poland; 3 Jagiellonian Centre for Experimental Therapeutics (JCET), Jagiellonian University, Kraków, Poland; 4 Department of Chemical Physics, Faculty of Chemistry, Jagiellonian University, Kraków, Poland

**Keywords:** Particulate matter, Inorganic residues of particulate matter, Protein oxidative modification, OVA immunogenicity, Autoimmunity

## Abstract

Exposure to air particulate matter (PM) is linked to the blood oxidative stress and systemic inflammation. The aim of this study was to elucidate whether oxidative PM modification of ovalbumin (OVA), the major antioxidant serum protein, may alter its antigenicity and/or immunogenicity. Ovalbumin was exposed via dialysis to the standard urban PM (SRM 1648a) or to PM with removed organic content (encoded as LAP). Both structural changes and biological properties of PM-modified OVA were measured. T lymphocytes and dendritic cells (the major antigen-presenting cells) isolated from C57BL/6 and OT-II (323–339 epitope) OVA-specific T cell receptor (TCR)-transgenic mice were used to test the effect of PM on OVA immunogenicity. The immunogenicity of both SRM 1648a and LAP-modified OVA was significantly higher than that of control OVA, as measured by the epitope-specific T cell proliferation and interferon γ production by the stimulated cells. This effect was associated with mild oxidative changes in the carrier molecule outside the structure of the OVA epitope and with increased resistance to proteolysis of PM-modified OVA. Interestingly, dendritic cells showed enhanced capacity for the uptake of proteins when the cells were cultured with PM-modified OVA. Our results suggest that the enhanced immunogenicity of PM-modified OVA is not associated with altered antigenicity or antigen presentation. However, it may result from slower degradation and longer persistence of modified antigens in dendritic cells. Whether this phenomenon is associated with enhanced risk prevalence of autoimmune diseases observed in the areas with high urban PM pollution needs to be explained.

## Introduction

Exposure to air particulate matter (PM) is linked to aggravation of respiratory, cardiovascular, inflammatory and autoimmune diseases (Arias-Pérez et al. 2020; Hamanaka and Mutlu 2018). It has been reported that PM is responsible for enhanced oxidative stress and inflammation (Gawda et al. 2017; Mazzoli-Rocha et al. 2010), especially PM nanoparticles have a great potential for penetrating the respiratory system. Further diffusion to the pulmonary alveoli is possible and a fraction of these fine particles can enter the blood circulation where their exposure to blood components is well documented (Alemayehu et al. 2020; Nemmar et al. 2002). Therefore, PM can either stimulate pulmonary inflammatory cells to produce reactive oxygen species or directly modify the structure of serum proteins, particularly albumin (Chiang et al. 2013; Preston et al. 2020). Elucidation of the effect that air pollutants have on the structural modification and biological activity of albumin is of great importance, as albumin is the major antioxidant serum protein. Recently, we have shown that upon exposure to aqueous extracts of urban airborne particulate matter (SRM 1648a), albumin does not aggregate and while its structure is only slightly changed, it is loaded with a variety of metal ions (Al, Fe, Zn, Pb). Such form of albumin might remain in the circulatory system and interfere with other proteins by dissemination of accumulated metal ions in an uncontrolled way (Mazuryk et al. 2020). Furthermore, it has been shown that oxidative modification of bovine serum albumin (BSA) caused by diesel exhaust particles (DEP) results in the generation of the oxidized form of BSA that could lead to oxidative stress and activation of inflammation (Chiang et al. 2013). Thus, PM exposure, via the modification of serum albumin, may either induce or exacerbate systemic inflammation. While the impact of PM on albumin immunogenicity remains unclear, the oxidative modification of ovalbumin (OVA) by reactive oxygen species (ROS) is well documented (Bruschi et al. 2013; Olszowski et al. 1996). The oxidative modification of OVA induced by HOCl-dependent chlorination of target amino acids results in ovalbumin accelerated uptake and processing by antigen-presenting cells (APCs) and finally more effective stimulation of OVA-specific T helper cells (Prokopowicz et al. 2010).

Herein, the impact of standard PM representing urban pollution on the structure and biological functions of OVA was evaluated. Key goals of our research on the interaction of PM with OVA were as follows: (1) to investigate the effects of particle exposure on OVA immunogenicity, i.e., ability to stimulate OVA-specific T cell response; (2) to clarify OVA (structural/chemical) modification induced by PM (SRM 1648a or carbon-reduced SRM 1648a encoded as LAP). SRM 1648a, obtained from the National Institute of Standards and Technology, is considered to be the quality reference material for air pollutants with known elemental composition provided with the certificate of the material. The size of the particles varies from 0.2 to over 100 μm with a predominance of particles with a size of 10–20 μm (Wise 2012). LAP, oxygen plasma-treated SRM 1648a, was used as a reference particulate matter material with decreased organic carbon content to compare the difference between the original PM and the inorganic fraction of PM (Mikrut et al. 2021).

Ovalbumin has been chosen as the representative target protein because its structure is well known, including the structure of epitopes recognized by OVA-specific T cells. Moreover, the experimental model to test the immunogenicity of OVA in vitro is available (Robertson et al. 2000).

## Materials and Methods

### PM Preparation

Standard Reference Material 1648a (encoded as SRM 1648a) was supplied by the National Institute of Standards and Technology (USA). Particulate matter samples with removed organic content (< 2% of organic carbon, < 1% of nitrogen, encoded as LAP) were obtained by the treatment of SRM 1648a with a low-temperature plasma for 2 h according to the published procedure (Mikrut et al. 2018). All other reagents were of reagent grade. All suspensions were prepared using ultrapure water.

### Protein Preparation

Albumin from chicken egg white (OVA; Sigma-Aldrich, Germany) dissolved in phosphate-buffered saline (PBS) at 4 mg/ml concentration was placed in Pur-A-Lyzer Midi Dialysis Kit (Sigma-Aldrich, Germany) device with a cutoff at 3.5 kDa. The dialysis was carried out for 6 h at 4 °C in PBS (pH 7.4; Corning, USA) alone (control OVA) or PBS containing 2 mg/ml suspensions of SRM 1648a or LAP (PM-modified OVA), followed by dialysis against a fresh portion of PBS for another 6 h (4 °C). PBS was changed every hour. This procedure was performed under sterile conditions. After dialysis, OVA was collected by reversed centrifugation and either used immediately or frozen in small aliquots for further analysis. The concentration of OVA after the dialysis was measured spectrophotometrically and was typically in the range 3.67 ± 0.02 mg/ml.

### Mice

Inbred male C57BL/6 (wild-type, WT) and OT-II T cell receptor (TCR)-transgenic mice were bred in the Animal Breeding Unit, Department of Immunology of Jagiellonian University College of Medicine, Krakow. OT-II CD4^+^ T cells express transgenic OVA-specific αβ-TCRs. Mice were housed five to six per cage and maintained under clean conventional conditions with free access to standard rodent diet and water. Mice were used at 8–10 weeks of age. All animal procedures were in agreement with the guidelines from Directive 2010/63/EU of the European Parliament on the protection of animals used for scientific purposes.

### Cell Preparation

Lymph nodes and spleens collected from naive OT-II OVA-specific TCR-transgenic mice were squeezed through the cell strainer to obtain single cell suspension. Whole lymph node cell population was used for the culture in vitro (as described below) or the cells (from lymph nodes and spleens) were incubated with anti-mouse CD4 MicroBeads (Miltenyi Biotec, USA) 15 min at 4 °C and after washing CD4^+^-enriched T cells were isolated by magnetic activated cell sorting (MACS) on MS (or LS) MACS columns (Miltenyi Biotec, USA) according to the manufacturer’s guidance.

Spleens were collected from C57BL/6 mice to isolate CD11c^+^ dendritic cells (DCs). Tissue was digested at 37 °C, in RPMI-1640 (BioWhittaker™, Lonza, Switzerland) containing 10% fetal bovine serum (FBS; Biowest, France), 40 U/ml collagenase type I from *Clostridium histolyticum* (Sigma-Aldrich, Germany) and 30 U/ml DNase from bovine pancreas, grade I (Roche/Sigma-Aldrich), 2 × 30 min. Cells were washed in RPMI-1640 containing 10% FBS, and then in PBS containing Mg^2+^, Ca^2+^ and 2% BSA (Sigma-Aldrich, Germany). Next, cells were incubated with anti-mouse CD11c MicroBeads (Milenyi Biotec, USA) 15 min at 4 °C and after washing CD11c^+^-enriched DCs were manually isolated in the strong magnetic field on MS (or LS) MACS columns (Miltenyi Biotec, USA).

### T Cell Proliferation Assay In Vitro

Cells were cultured in vitro in 5% CO_2_ atmosphere at 37 °C, in RPMI-1640 medium (BioWhittaker^™^, Lonza, Switzerland) containing 5% FBS (Biowest, France) supplemented with 25 mM HEPES (Gibco, Thermo Fisher, UK), 2 mM L-glutamine (Biowest, France), 0.05 mM 2-mercaptoethanol (Gibco, Thermo Fisher, UK), and 5 mg/ml gentamicin (KRKA, Slovenia). Cells were seeded in 0.2 ml in 96-well plates (Falcon, Corning Inc., USA), with lymph node cells at 2 × 10^5^/well and isolated CD11c^+^ DCs at 1 × 10^4^/well with CD4^+^ T cells at 1 ×  × 10^5^/well (the ratio DC:T cells = 1:10), and cultured for 48 h at 37 °C in 5% CO_2_ atmosphere, in the presence of 100 μg/ml control (dialyzed against PBS) or PM-modified OVA. All groups were investigated in triplicate. Cell culture supernatant was harvested and stored at – 20 °C for cytokine (interferon (IFN)-γ) assay. Fresh medium was added and cells were pulsed with 3H-thymidine (1 μCi/well, Polatom, National Centre for Nuclear Research, Poland) and cultured further for 16–18 h. Then cells were harvested onto a glass fiber filter (Perkin Elmer, USA) using Filtermate Harvester (Packard Bioscience/Perkin Elmer, USA). Incorporation of radioactive 3H-thymidine was evaluated using microplate scintillation counter (Microbeta Wallac TriLux Scintillation and Luminesce Counter; PerkinElmer, USA).

### Cytokine ELISA Assay

Mouse IFN-γ ELISA Ready-SET-Go! Kit (Invitrogen, Thermo Fisher Scientific, USA) was used and the assay was performed according to the manufacturer’s instructions. Shortly, Costar EIA/RIA plates (Corning Incorporated, USA) were coated with anti-mouse cytokine antibodies overnight at 4 °C. After blocking the plates, serial dilutions of mouse IFN-γ standards and diluted cell culture supernatants were applied to the plates and incubated overnight at 4 °C. Then biotin-conjugated anti-IFN-γ antibodies were added for 1 h (room temperature), followed by avidin-HRP and TMB as detection reagent. The optical density was read at 450 nm.

### Analysis of PM-Modified OVA

#### Measurement of Sulphhydryl Level

The content of free –SH groups in dialyzed protein samples was estimated spectrophotometrically by the reaction of 90 µM dialyzed OVA with 0.9 mM Ellman’s reagent 5,5’-dithiobis(2-nitrobenzoic acid) (DTNB) and 2 mM cystamine, using a molar extinction coefficient of 13,600 M^–1^ cm^–1^ at 412 nm.

#### Assays of Carbonyl Group Content

A solution of 10 mM 2,4-dinitrophenylhydrazin (DNPH) in 2 M HCl was added to dialyzed ovalbumin samples to give a final protein concentration of 1 mg/ml. The solutions were mixed vigorously for 1 h, then precipitated with 20% trichloroacetic acid (TCA) and placed on ice for 10 min. After this time, the solutions were centrifuged (3000*g*, 10 min), the supernatants were discarded and the protein pellets were washed three times with 3 ml portions of ethanol/ethyl acetate mixture (1:1, v/v) to remove any free DNPH. The samples were then resuspended in 6 M guanidine (pH 2.3). The carbonyl content was determined from the absorbance at 370 nm using a molar absorption coefficient of 22,000 M^–1^ cm^–1^.

#### OVA Oxidative Modification

Dialyzed OVA was reduced with 10 mM dithiothreitol in 50 mM ammonium bicarbonate at 37 °C for 45 min while shaking (500 rpm). Then, the prepared samples were alkylated with 20 mM iodoacetamide for 30 min and treated with 1.8 µg trypsin in 50 mM ammonium bicarbonate overnight at 37 °C. The digestion was stopped by adding 1% formic acid. The samples were centrifuged for 5 min, 12 000 rpm at 4 °C, and the supernatants were analyzed with high-resolution UPLC–MS/MS (UltiMate 3000 RS, Q Exactive Plus, Thermo Fisher Scientific, USA). Typically, 5 µl of the sample was injected into a UPLC–MS/MS in an ACQUITY UPLC HSS T3 1.8 µm column. The separation was run with the gradient mode of Phase A (0.1% formic acid in water) and Phase B (0.1 formic acid in acetonitrile) at a flow rate of 0.2 ml/min. For qualitative analysis, full MS/dd-MS^2^data acquisition mode was used. The obtained spectra were processed in Proteome Discoverer 2.4 and BioPharma Finder 3.1 (Thermo Fisher Scientific, USA) software to identify the modifications of protein. In Proteome Discoverer 2.4, proteins with modifications were identified based on comparison with data stored in the most popular database SwissProt, searched with Mascot algorithm. In BioPharma Finder 3.1 software, modifications were identified based on the comparison with a manually created sequence of the OVA. The created sequence is listed below:

 > sp|P01012|OVAL_CHICK Ovalbumin OS = Gallus gallus OX = 9031 GN = SERPINB14 PE = 1 SV = 2.

MGSIG AASME FCFDV FKELK VHHAN ENIFY CPIAI MSALA MVYLG AKDST RTQIN KVVRF DKLPG FGDSI EAQCG TSVNV HSSLR DILNQ ITKPN DVYSF SLASR LYAEE RYPIL PEYLQ CVKEL YRGGL EPINF QTAAD QAREL INSWV ESQTN GIIRN VLQPS SVDSQ TAMVL VNAIV FKGLW EKAFK DEDTQ AMPFR VTEQE SKPVQ MMYQI GLFRV ASMAS EKMKI LELPF ASGTM SMLVL LPDEV SGLEQ LESII NFEKL TEWTS SNVME ERKIK VYLPR MKMEE KYNLT SVLMA MGITD VFSSS ANLSG ISSAE **SLKIS QAVHA AHAEI NEAG**R EVVGS AEAGV DAASV SEEFR ADHPF LFCIK HIATN AVLFF GRCVS P.

Epitopes recognized by OT-II transgenic TCR, amino acids number 323–339 are marked in bold.

#### Monitoring Amino Groups in OVA

Dialyzed OVA was diluted with 0.2 M borate buffer, pH 9, containing 0.1 M EDTA, and 1 mM 2,4,6-trinitrobenzenesulfonic acid solution (TNBS) was added. Samples were mixed well and incubated at 37 °C for 2 h. Then 10% sodium dodecyl sulfate (SDS) in 1 M HCl was added to each sample to stop and stabilize the reaction. The absorbance at 335 nm was measured and the concentration of amino groups in OVA was determined using a standard curve constructed with glycine.

#### Aggregation of OVA

Aggregation of OVA was investigated using FPLC (Bio-Rad, USA). Dialyzed OVA samples were injected into Superdex 200 10/300 GL column and absorbance at 280 nm was monitored. Separation was conducted by isocratic elution with 50 mM Tris buffer, pH 7.4, containing 100 mM NaCl at 8 °C.

#### Fragmentation of OVA

The sensitivity of OVA to different proteolytic enzymes (trypsin, protease K, cathepsin S) was investigated. Ovalbumin was diluted up to the 2 mg/ml concentration with 50 mM Tris buffer, pH 8, containing 0.1 M EDTA and additionally 5 mM CaCl_2_ (in case of proteinase K). Enzymes were added to a final enzyme:protein ratio of 1:30 (w/w). Samples were incubated at 37 °C for 24 h, then heated up to 90 °C to stop the reaction. 15% SDS-PAGE was used to separate the digested OVA protein fragments. Each sample was measured in duplicate, and the full experiment run in triplicate to increase the confidence in the obtained results.

All chemicals used in section “Analysis of PM-modified OVA” were from Sigma-Aldrich, Germany.

#### Differential Scanning Fluorimetry (nanoDSF)

Thermal unfolding was analyzed as the increase of intrinsic fluorescence intensity of the protein using a nanodifferential scanning fluorimetry instrument (Prometheus Panta, NanoTemper). This technique detects small changes in the fluorescence of tryptophan upon folding or unfolding in a label-free manner as a function of temperature. The samples were excited at 280 nm and fluorescence was detected at 330 and 350 nm. The temperature was ramped at 1 °C/min from 25 to 95 °C. The ratio of 350/330 nm was used for data analysis. Protein melting temperatures (*T*_m_) as well as the temperatures of the onset of unfolding (*T*_onset_) were determined from the thermal unfolding curve with the software PR.Panta Analysis. Measurements were taken using 10 µl sample volume per measurement in standard grade capillaries. Each sample was measured in triplicate and the full experiment run in duplicate to ensure reproducibility.

### Raman Imaging of DCs

Dendritic cells, CD11c^+^ isolated from mouse spleen (as described above), were incubated for 4 h at 37 °C in 5% CO_2_ atmosphere, in RPMI-1640 medium containing 5% FBS, in the presence of 100 μg/ml control (dialyzed against PBS) or PM-modified OVA. After incubation, cells were washed three times with PBS and fixed with 4% formaldehyde.

Raman imaging of fixed cells was performed using a confocal Raman microscope WITec Alpha 300 (Ulm, Germany) equipped with a CCD detector (Andor Technology Ltd, Belfast, Northern Ireland). For single cell measurements, a 40 × water dipping objective (Zeiss W Plan-Apochromat 40 × , NA = 1, Oberkochen, Germany) was used. Before the measurements, the cell suspension was centrifuged and then approximately 400 µl of cell suspension was deposited onto the CaF_2_ slide (Crystran LTD, Poole, UK, Raman grade) and placed under the objective of the microscope. Raman spectra were acquired with an excitation laser wavelength at 532 nm. Data were collected with an acquisition time per spectrum of 0.5 s, sampling density of 1 μm, and spectral resolution of 3 cm^−1^. For each sample, around 30–40 cells were imaged, and the experiment was done in triplicate.

Spectral data post-processing was conducted using Project FIVE 5.1 Plus software (WITec GmbH, Germany). Spectral pre-processing included removal of artifacts from cosmic radiation, subtraction of background contributions and residual autofluorescence (polynomial fitting, third order). The mean spectra of a single cell were obtained by averaging all cellular spectra in a hyperspectral image using an algorithm written in the Python language based on the calculated values of Pearson’s coefficient between single cellular spectra in a hyperspectral image and a reference spectrum of cells. The mean single cell spectra were next vector normalized in the spectral range of 3050–400 cm^–1^. Integral intensities of 2930 cm^–1^ Raman band were calculated using OPUS 7.2 software (Bruker Optik GmbH, Ettlingen, Germany). The integrals under the 2930 (I_2930_; proteins) band were calculated in the following spectral ranges: 2914–3010 cm^–1^.

### Statistical Analysis

Significant differences among tested samples were determined by one-way analysis of variance (ANOVA) using STATISTICA software. The probabilities of *p* < 0.05 were considered statistically significant and marked on relevant figures (*p < 0.05).

## Results

### Antigen-Specific T Cell Proliferation Assay

#### The Effect of PM, SRM 1648 or LAP

**Fig. 1:**
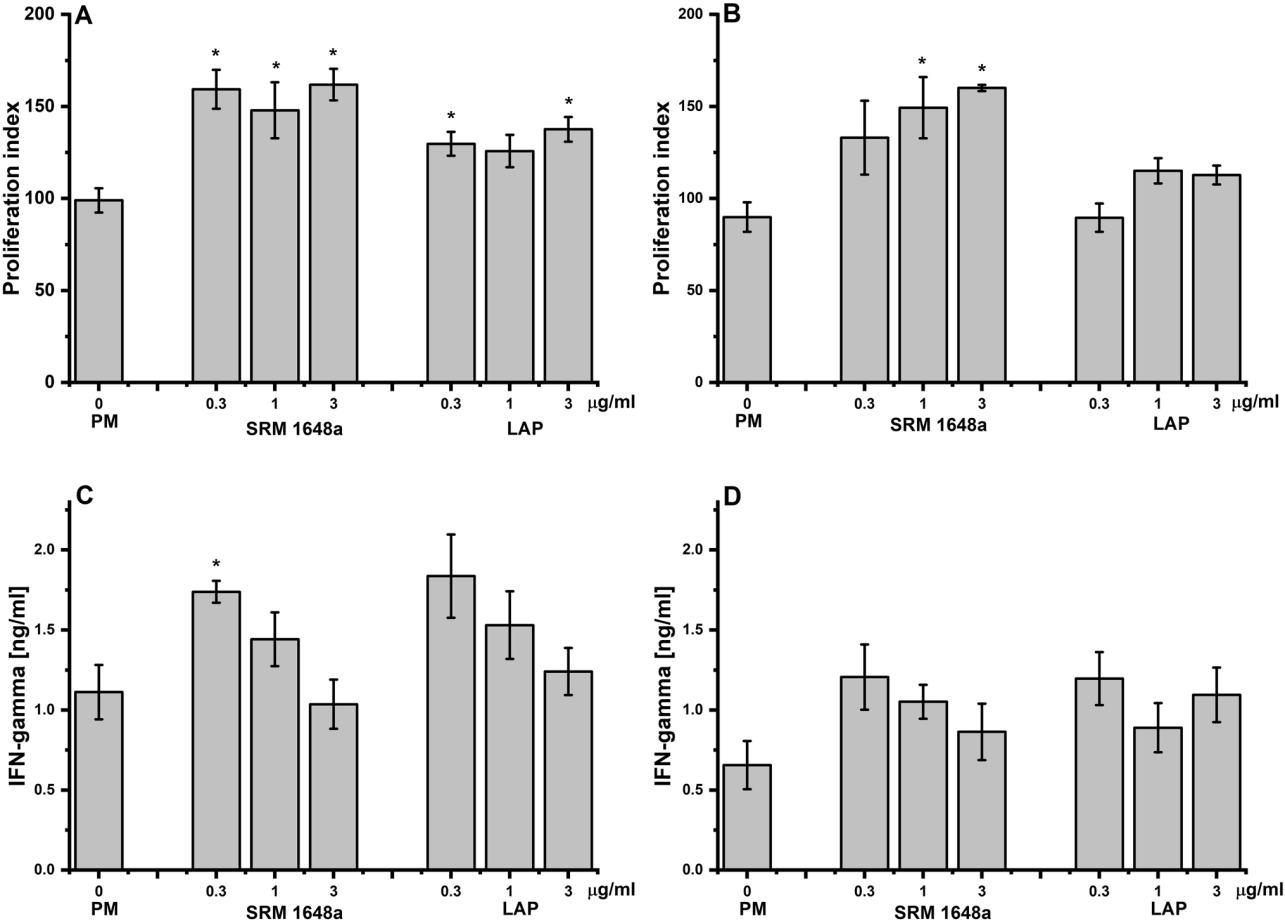
WT CD11c^+^ DCs and OT-II OVA-specific TCR-transgenic CD4^+^ T cells were cultured with (**A**, **C**) native OVA (100 μg/ml) or (**B**, **D**) OVA 323–339 peptide (1 μg/ml) in the presence of PM – SRM 1648 or LAP (0.3, or 1, or 3 μg/ml). After 48 h, the cell culture supernatant was collected and then fresh medium and 3H-thymidine (1 μCi) were added to the culture. Incorporation of 3H-thymidine is shown as proliferation index calculated according to the formula: CPM Ag^+^/CPM Ag^–^ = counts per minute of cells stimulated with antigen/CPM of cells cultured without antigen; antigen = native OVA (**A**) or OVA 323–339 peptide (**B**). IFN-γ (ng/ml) in 48 h cell culture supernatant, OVA stimulated (**C**), or OVA 323–339 peptide stimulated (**D**) were measured by ELISA. Results are expressed as a mean of the measurements of cell culture triplicate ± SEM. Data shown are representative of three independent experiments. *p < 0.05 denotes statistically significant differences between control group (cultured without PM samples) and the tested groups (cultured in the presence of PM samples)

To examine the effect of airborne PM (SRM 1648a, LAP) on antigen-specific T cell response, WT (C57BL/6) dendritic cells (CD11c^+^) and OT-II OVA-specific TCR-transgenic CD4^+^ T cells were stimulated in vitro with native OVA (100 µg/ml) or OVA 323–339 peptide (1 µg/ml) in the presence of PM samples at various concentrations (0.3, 1.0, 3.0 µg/ml). Proliferation rates of T cells is shown as proliferation index defined as the ratio of counts per minute (CPM) of antigen (OVA)-stimulated cells to CPM of unstimulated cells. As shown in Fig. 1A, the proliferative response of T cells to OVA was significantly enhanced when PM samples (SRM 1648a or LAP) were present, regardless of the concentration of PM introduced into the culture. Similarly, proliferation of T cells in response to OVA 323–339 peptide was increased in the presence of PM, especially SRM 1648a. The effect of LAP on OVA 323–339 peptide-induced T cell proliferation was less prominent (Fig. 1B). These data suggest that intake of the antigen by CD11c^+^ DCs (APC) in the presence of SRM 1648a was accelerated (facilitated). Interestingly, IFN-γ production by T cells in response to OVA stimulation was significantly augmented only by low concentration (0.3 µg/ml) of SRM 1648a (Fig. 1C). PM (SPRM 1648 s, LAP) in higher concentration to a lesser extent influenced the production of IFN-γ induced by OVA (Fig. 1C) or OVA 323–339 peptide (Fig. 1D), and observed differences were not significant statistically.

### The Effect of PM-Modified OVA

**Fig. 2:**
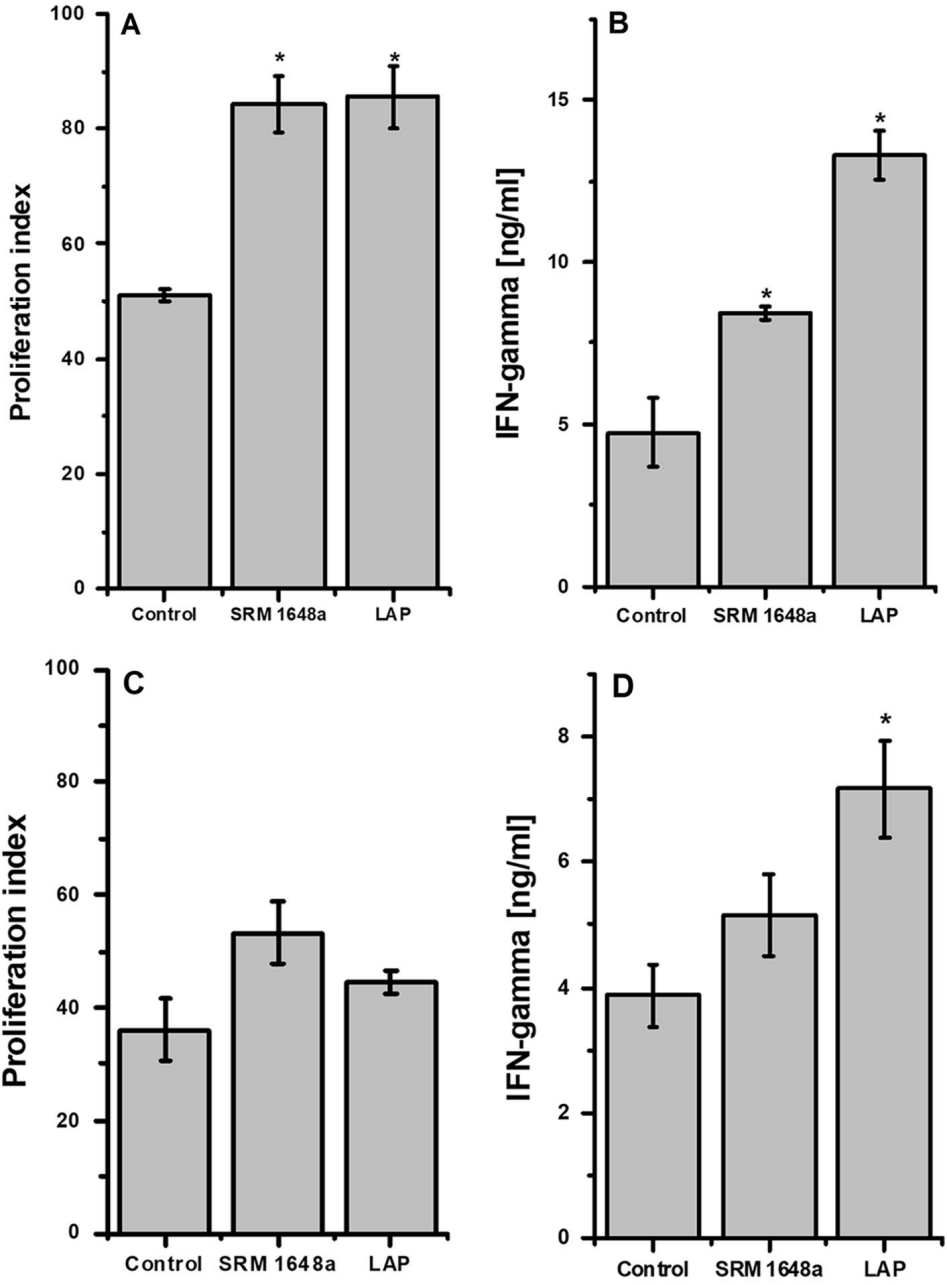
Lymph node cells (**A**, **B**) or CD11c^+^ DCs with OT-II TCR-transgenic CD4^+^ T cells in the ratio 1:10 (DC:T cells) (**C**, **D**) were cultured for 48 h in the presence of OVA (100 μg/ml) – control (dialyzed against PBS) or modified by PM via dialysis against SRM 1648a (2 mg/ml) or LAP (2 mg/ml). IFN-γ (ng/ml) (**B**, **D**) in cell culture supernatant was measured by ELISA. Proliferation of T cells (**A**, **C**) was measured by 3H-thymidine incorporation and the results are shown as proliferation index calculated according to the formula: CPM OVA^+^/CPM OVA^–^. Results are expressed as a mean of the measurements of cell culture triplicate ± SEM. Data shown are representative of three independent experiments. *p < 0.05 denotes statistically significant differences between the control group (OVA dialyzed against PBS) and the tested groups (OVA dialyzed against PM samples)

To examine the immunogenicity of PM-modified antigen, lymph node cells from OT-II OVA-specific TCR-transgenic mice were stimulated in vitro with control or PM-modified OVA. As shown in Fig. 2, the proliferation rates of cells cultured in the presence of OVA modified by SRM 1648a or LAP were significantly higher and those cells produced significantly more IFN-γ compared to cells stimulated with control OVA. Although the cell proliferation rates in response to SRM 1648a- or LAP-modified OVA were comparable (Fig. 2A), cells cultured in the presence of LAP-modified OVA produced more IFN-γ (Fig. 2B).

To further clarify the effect of protein PM modification on antigen-specific T cell response, DCs (CD11c^+^ enriched) isolated from WT mice (C57BL/6) were cultured in vitro together with OT-II TCR-transgenic T cells (CD4^+^ enriched) in the presence of SRM 1648a- or LAP-modified OVA. Proliferation of T cells in response to PM-modified OVA presented by CD11c^+^ cells was higher compared to control OVA (Fig. 2C). Cells showing enhanced proliferation in response to SRM 1648a- or LAP-modified OVA produced also more IFN-γ. The production of IFN-γ by T cells was significantly higher in response to OVA modified by LAP (Fig. 2D).

### Functional Group Modification in OVA

#### Effect of PM on the Sulfhydryl Group Content in OVA

**Fig. 3:**
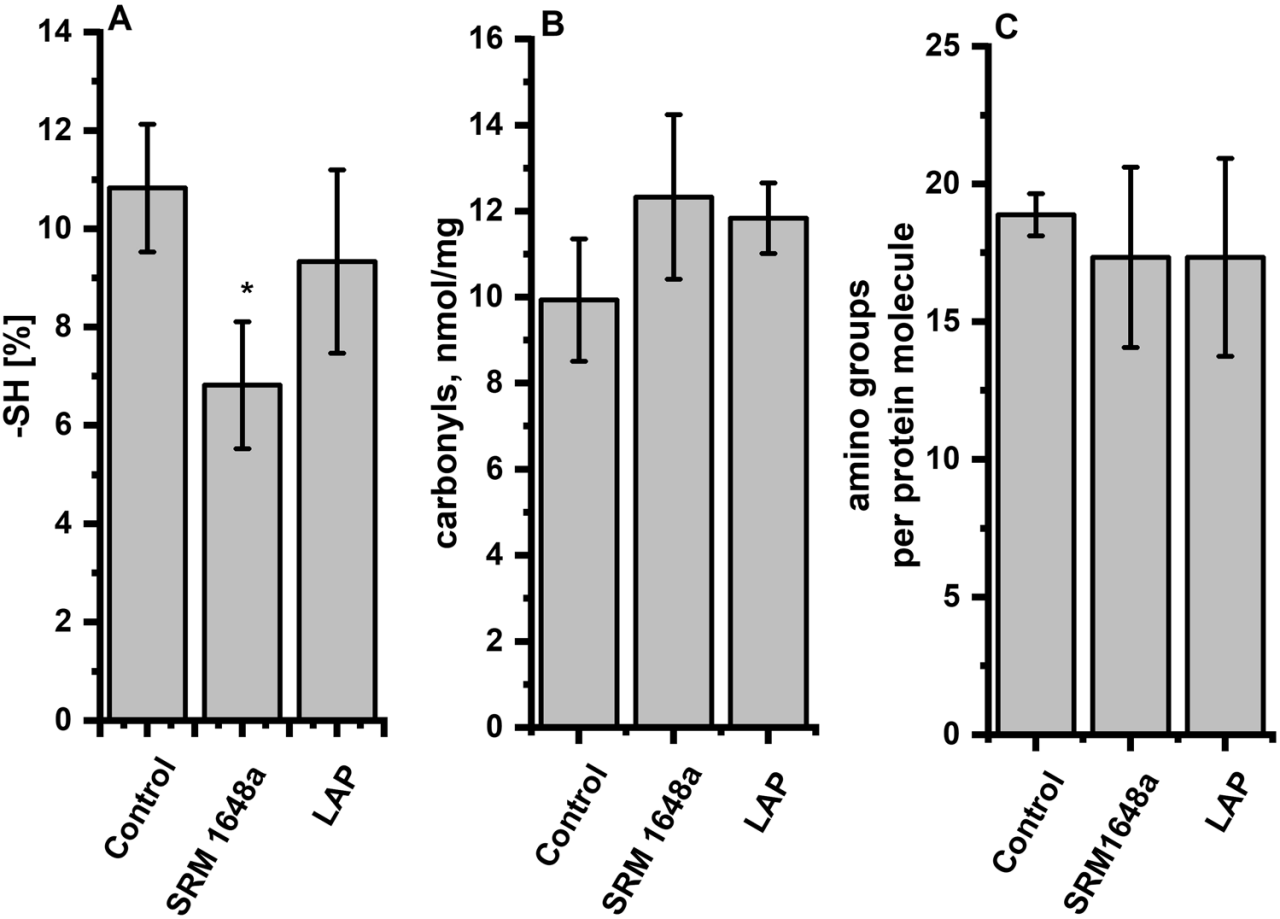
Functional modifications in ovalbumin (OVA; 4 mg/ml) dialyzed against PBS (control) or PM (SRM 1648a or LAP, 2 mg/ml). The assessment of free thiol groups (**A**, *p < 0.05), carbonyl groups (**B**) and amino groups (**C**) determined in dialyzed ovalbumin presented per protein molecule. The results are presented as mean values ± SEM from a minimum of three independent experiments

To check whether PM (SRM 1648a or LAP) can cause permanent changes in the availability of free thiols in OVA, dialyzed OVA was analyzed using DTNB, a common free –SH assay reagent. As shown in Fig. 3A, considerable free thiol depletion in OVA after the dialysis against PM suspensions was determined. Ovalbumin contains four free –SH groups buried in the protein core, whose availability is limited under physiological conditions. Dialysis against SRM 1648a resulted in a significant decrease in the mean free thiol level in the protein, whereas the effect of LAP was much weaker. This indicates that under investigated conditions, ovalbumin’s cysteine became unavailable.

#### Effect of PM on the Carbonyl Group Content in OVA

Carbonyl groups formation is a common marker of protein oxidation. To check whether the dialysis of OVA in the presence of PM (SRM 1648a, LAP) can cause amino acid residues’ carbonylation, the dialyzed protein was analyzed using 2,4-dinitrophenylhydrazine. Results presented in Fig. 3B indicate that the amount of carbonyl groups slightly increased in PM-treated OVA. Both variants of PM, SRM 1648a and LAP induced similar increase in the amount of carbonyl group in dialyzed protein samples. Metal-catalyzed oxidation usually happens when reduced metal ions such as Fe^2+^ or Cu^+^ are engaged in the redox reaction. In the presence of molecular oxygen, highly reactive hydroxyl radical (HO^∙^) forms in the Fenton-like reaction catalyzed by copper or iron ions (Ugur and Gronert 2017). This effect can be boosted in the presence of H_2_O_2_ and accelerated with higher metal concentration (Krumova et al. 2009). However, since dialysis was conducted under aerobic conditions, participation of reduced forms of iron or copper (Fe^2+^ or Cu^+^) seems to be less likely, which is in agreement with our observations.

#### Effect of PM on the Amount of Free Amino Groups Content in OVA

The content of free amino groups in OVA was assessed using TNBS, which reacts with ε-amino groups in proteins and the absorption spectra of the produced complex can be measured (Sashidhar et al. 1994).

Ovalbumin contains 20 lysine residues per protein, which can be quantified with TNBS. The number of free amino groups was measured for OVA dialyzed in the presence of PMs. The obtained results (Fig. 3C) revealed a slight decrease in the amount of free amino groups in OVA dialyzed against both SRM 1648a and LAP. The decrease in the number of free amino groups might be attributed to the complexation reactions of Cu(II) with L-lysine and biogenic amines. Lysine may coordinate copper(II) ions with the potential sites of biocoordination interactions at oxygen atoms from the carboxyl group and nitrogen atoms from the α and ω amine. The addition of Cu(II) can reduce the amount of free amino groups either directly or by the formation of polyamine ternary complexes (Bregier-Jarzebowska et al. 2019).

### The Identification of Oxidative Modification of Dialyzed OVA

To identify the modifications of dialyzed OVA, UPLC–MS/MS analysis was performed. The obtained spectra were processed in Proteome Discoverer 2.4 and BioPharma Finder 3.1 software to identify the amino acid modifications. The list of identified modifications is presented in Table 1.

**Table 1:** The list of modifications identified by UPLC–MS/MS of dialyzed ovalbumin with regard to the position of modified amino acid residue in the protein

Modification	SRM 1648A	LAP	Control
Carbamidomethylation	C11, C31, C74, C121, C368	C11, C31, C74, C121, C368	C11, C31, C74, C121, C368
Oxidation	M9, M36, M197, M212	M9, M36, M197, M274	M9, M197
Carbonylation	P32	–	–

The obtained results indicate that the dialysis of OVA in the presence of PM leads to additional oxidation and carbonylation of the protein.

Cysteine carbamidomethylation is a static modification due to a reaction with iodoacetamide, which was introduced into the sample preparation step to obtain higher sequence coverage enabling better identification of the other modifications. This modification was identified for all cysteine residues and prevented the oxidation of cysteine.

As methionine oxidation is a common modification occurring during tryptic digestion, it was identified in control samples for the residues M9 and M197. However, when looking at the results obtained for the protein after the dialysis against PM, a significant increase in the number of oxidized methionine residues can be seen. In PM-treated OVA, the additional modifications of M36, M212 and M274 occurs versus the control. In addition, the amount of oxidized methionine M197 in the protein samples after dialysis against PM was considerably higher than in control ovalbumin (dialyzed against PBS only). The results indicate higher susceptibility of OVA dialyzed against metal compounds to methionine oxidation during trypsin treatment. Another type of specific amino acid modification was the carbonylation of proline residues identified for protein after the dialysis against SRM 1648a (P32). Dialysis of OVA in the presence of PM and carbon-reduced PM resulted in analogous carbamidomethylation and oxidation of the amino acid residues. The only differences between after SRM 1648a and LAP treatment of the protein were found in the various methionine groups oxidation (M212 and M274) and in the carbonylation of P32 residue. The lack of clear and significant differences between the two variants of PM may indicate the negligible effect of organic components in the process of modifying the protein structure.

### OVA Aggregation

**Fig. 4:**
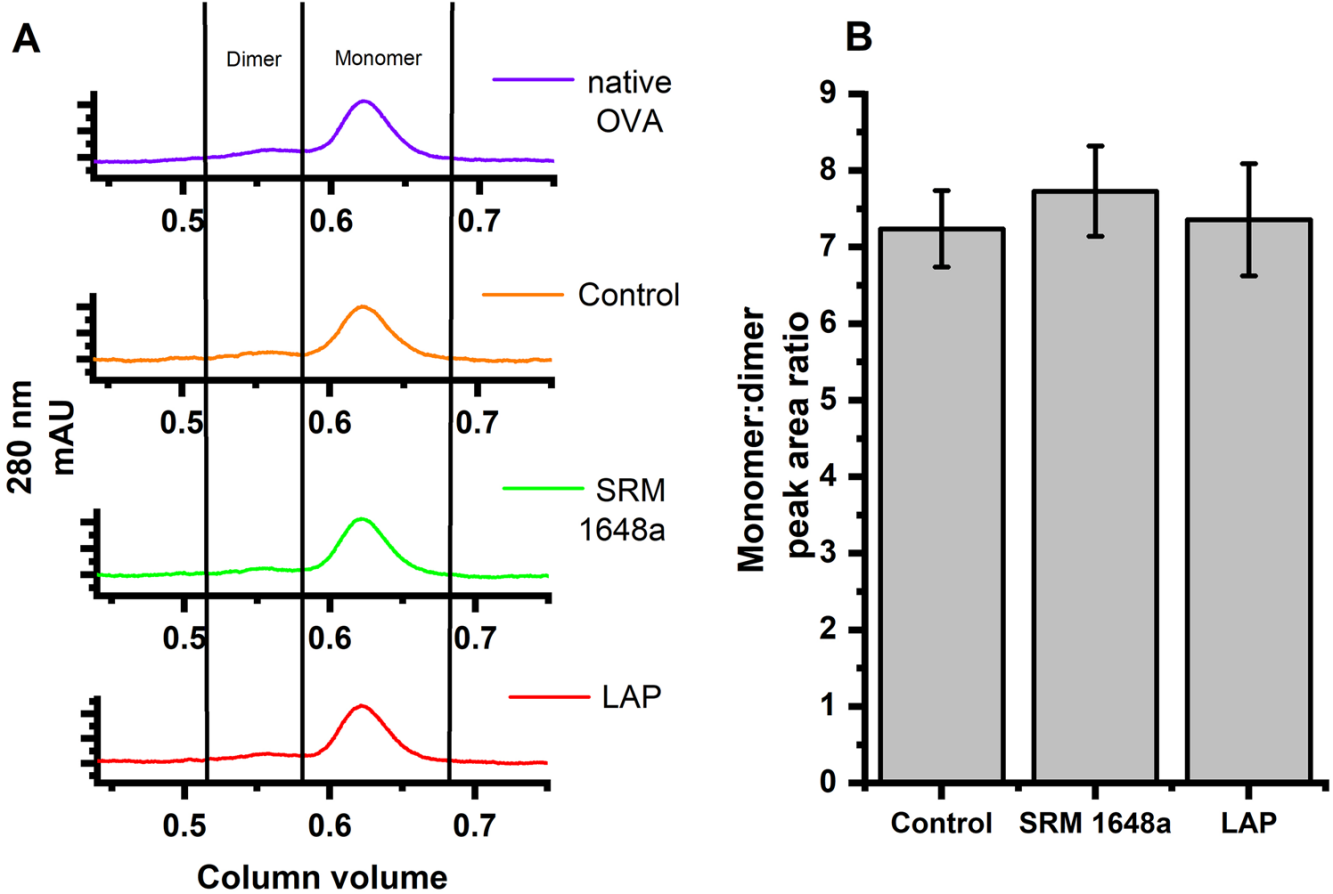
Representative chromatograms of the dialyzed ovalbumin (**A**). Ratio of the monomeric to dimeric (M:D) fractions (peaks area) of ovalbumin (OVA, 4 mg/ml) dialyzed against PBS (control) or PM suspensions (SRM 1648a or LAP, 2 mg/ml) (**B**)

The aggregation of ovalbumin dialyzed in the presence of PM was monitored by FPLC chromatographic separation at 280 nm (Fig. 4A). The monomeric fractions were assigned to a column volume range of 0.6–0.7, and the dimeric fractions to a column volume range of 0.5–0.6. Area ratios under the absorbance curves for monomeric and dimeric fractions (M:D) were compared to determine the degree of protein aggregation (Fig. 4B). A slight increase in the M:D area ratio can be observed for the SRM 1648a-treated OVA which may indicate a minor dimer dissociation under this condition.

### Sensitivity of Dialyzed OVA to Proteolytic Enzymes

**Fig. 5:**
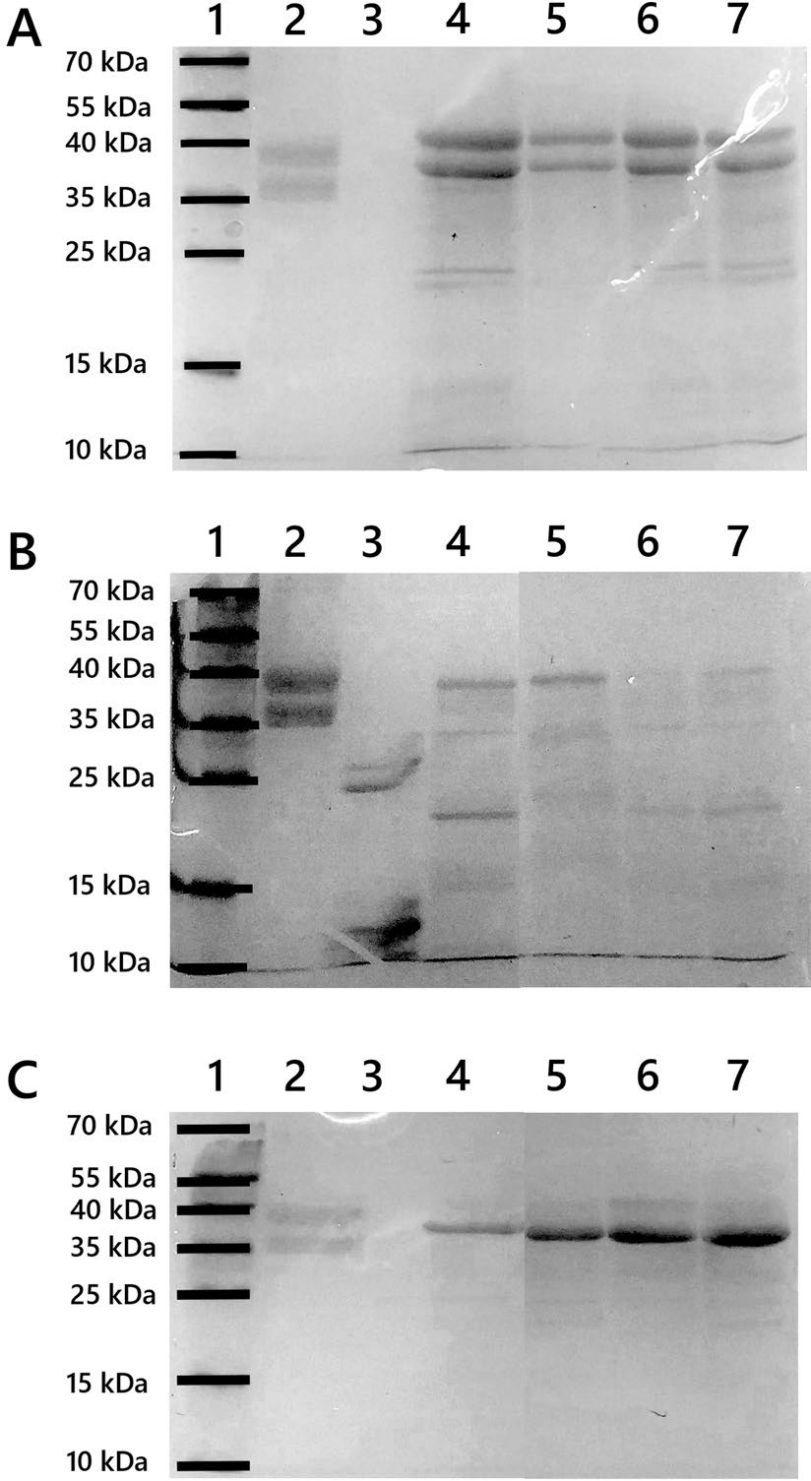
Representative images of SDS-PAGE analysis of dialyzed OVA digested by trypsin (**A**), proteinase K (**B**) and cathepsin S (**C**). Lanes: 1 – molecular weight protein standard, 2 – non-digested native ovalbumin, 3 – relevant proteolytic enzyme, 4 – digested native ovalbumin, 5 – digested LAP-modified OVA, 6 – digested SRM 1648a-modified OVA, 7 – digested control ovalbumin (dialyzed against PBS)

Dialyzed OVA samples were digested using several proteolytic enzymes to assess their sensitivity to digestion. Obtained peptide fragments were separated and analyzed by SDS-PAGE electrophoresis (Fig. 5). Several different proteases (trypsin, protease K, cathepsin S) were chosen to observe possible similarity of the behavior pattern of dialyzed OVA against proteolytic enzymes.

Native OVA is quite prone to trypsin digestion. As can be seen in Fig. 5A, treatment of OVA with trypsin resulted in two new fragmentation bands around 22 and 20 kDa (lane 4). The dialysis of OVA against PBS buffer alone (control, lane 7) or against SRM 1648a (lane 6) did not change the susceptibility of the protein to proteolysis with trypsin. OVA, dialyzed in the presence LAP, showed a quite different behavior upon trypsin digestion (lane 5). LAP-treated OVA was remarkably more stable against the protease cleavage; therefore, the bands around 20 and 22 kDa were not visible.

Protease K was found to be the most efficient enzyme to digest ovalbumin (Fig. 5B). Native protein (lane 4) is almost completely digested creating peptide patterns around 35, 21 and 15 kDa. A small fraction of the protein which remained undigested is localized around 40 kDa. Dialysis against PBS (control sample, lane 7) or SRM 1648 (lane 6) increased the sensitivity of the OVA toward the digestion, producing small barely visible cleaved fragments. Dialysis against LAP (lane 5) slightly reversed that process, indicating the positive effect of metal ions on the proteins sensitivity toward proteinase K digestion.

Digestion of the OVA with cathepsin S, the cysteine cathepsin predominantly found in APCs (DCs, macrophages) (Smyth et al. 2022) (Fig. 5C), revealed similar patterns for digestion of native OVA and OVA dialyzed against PBS or PM samples. The two bands from undigested protein (40 and 36 kDa, lane 2) almost completely disappeared resulting in the band around 38 kDa. Messy bands between 24 and 38 kDa were repetitive and slightly more visible in case of dialyzed OVA samples.

The obtained results are consistent and suggest smaller susceptibility of OVA dialyzed against PM (especially against LAP) toward proteolytic enzymes digestion. Higher resistance to enzyme treatment can be explained by possible structural changes caused by metal ions physically or chemically bound to protein upon samples dialysis. Importantly, all experiments were performed at pH > 7, the optimum value for these enzymes’ stability, that corresponds to the pH of cell cytoplasm.

### Thermal Stability of Dialyzed OVA

The thermal stability of the OVA was assessed to compare the unfolding behavior of the dialyzed OVA samples. The protein melting temperature *T*_m_ indicates the temperature where 50% of protein molecules exist in an unfolded state. As can be seen in Table 2, the melting temperatures *T*_m_ for native OVA and OVA samples dialyzed against PBS alone (control) or PM (SRM 1648 or LAP) did not differ significantly. However, the temperatures of unfolding onset *T*_onset_ showed that dialysis against PM induced an increase in the *T*_onset_ values, especially evident in OVA sample dialyzed against LAP. Larger onset of unfolding indicated that despite similar melting temperatures, OVA dialysis against PM (SRM 1648 or LAP) increased the thermal stability of the protein. Similar results were obtained in the case of the human serum albumin (Mazuryk et al. 2020).

**Table 2:** Thermal stability data for the native and dialyzed ovalbumin, including the melting temperature (*T*_m_) and the temperature of the onset of unfolding (*T*_onset_)

OVA	*T*_m_ [°C]	*T*_onset_ [°C]
Native	72.83 ± 0.70	60.57 ± 0.40
Control	73.93 ± 0.28	61.82 ± 0.76
SRM 1648a	72.22 ± 0.62	62.23 ± 0.54
LAP	72.27 ± 0.17	65.17 ± 0.10

### Raman Imaging of DCs

**Fig. 6:**
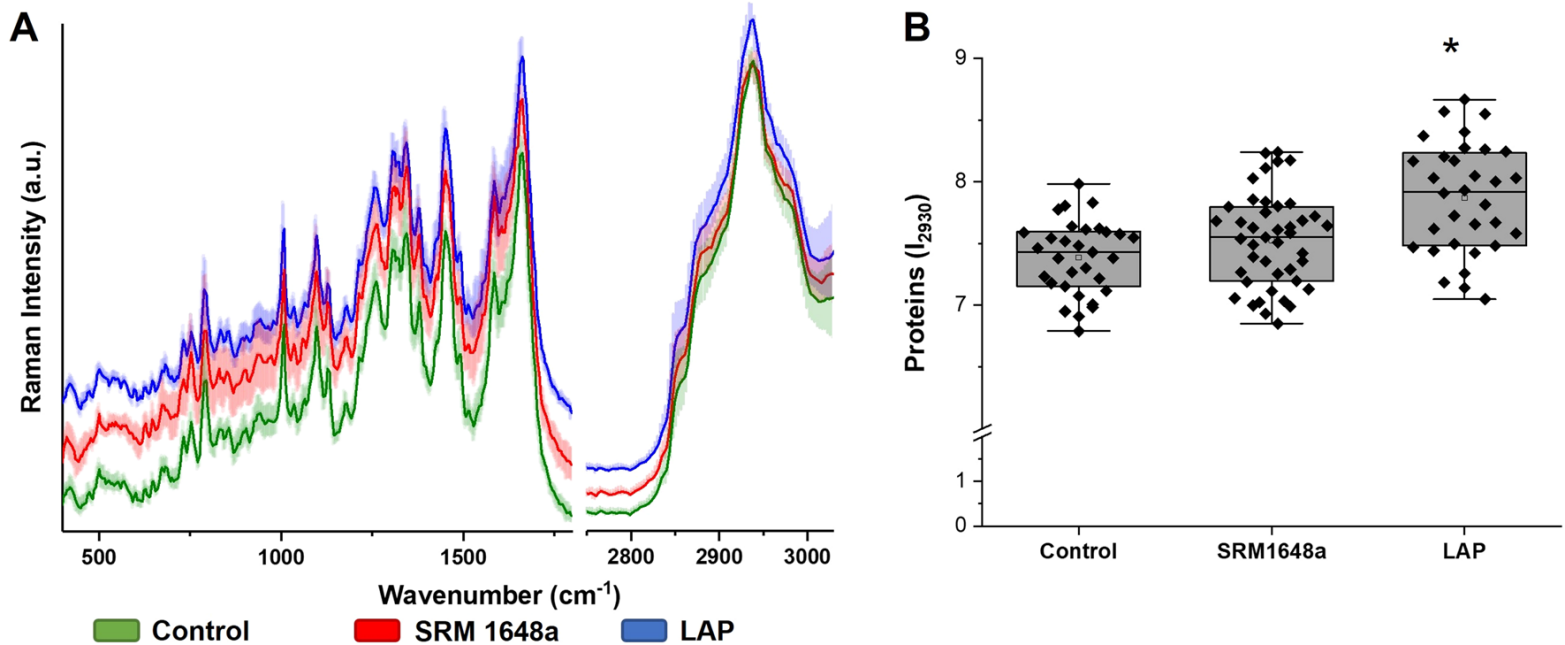
Average Raman spectra of the whole cells with the standard deviation (**A**) of cells incubated 4 h at 37 °C in 5% CO_2_ atmosphere, in RPMI-1640 medium containing 5% FBS, in the presence of 100 μg/ml control (dialyzed against PBS; green) or SRM 1641a-treated OVA (red) or LAP-treated OVA (blue) cells. Spectra were shown in the ranges of 400–1800 and 2750–3030 cm^–1^. Raman spectra in the fingerprint region (400–1800 cm^–1^) were multiplied by two for better visualization of spectroscopic features. The integral intensity ratio results (**B**) for Raman band at 2930 cm^–1^ calculated for control and treated cells are presented in box plots: median (horizontal line), SD (box), minimal and maximal values (whiskers). One-way ANOVA statistical analysis was used to evaluate significance of observed changes (*p < 0.05 vs control group)

To compare the protein content of DCs upon incubation with either control or PM-modified OVA, we employed Raman imaging technique (Majzner et al. 2020). In Fig. 6A, averaged spectra of all single cell mean Raman spectra of control group and cells treated with PM-modified OVA are displayed in the spectral ranges of 3030–2750 cm^–1^ and 1800–500 cm^–1^ with the standard deviation. Detailed analysis of Raman data revealed a statistically significant (*p* < 5%) increased total content of proteins in the LAP group vs control one (Fig. 6B). The slight increase for SRM 1648a group was not found to be statistically significant.

## Discussion

In the present study, the following issues concerning the influence of airborne PM (SRM 1648a, LAP) on the OVA-specific immune response were investigated: (i) the effect of PM on antigen (OVA or OVA 323–339 peptide) presentation by DCs and stimulation of T cells (Sethu et al. 2012); (ii) the effect of PM on OVA immunogenicity.

We have shown that for both native OVA and OVA 323–339 peptide, the OT-II TCR-transgenic epitope stimulated OT-II T cells more effectively in the presence of PM. OT-II transgenic mice have been commonly used as a model for studies on antigen-specific T cell responses in various contexts (Clemen et al. 2021; Krone et al. 2022). Our results exclude the effect of PM on antigen (Ag) processing, since OVA 323–339 peptide (epitope) presentation does not require any intracellular processing. The observed results may be explained by the enhanced internalization/endocytosis of both molecules (OVA protein and OVA 323–339 peptide) by APCs which would subsequently boost proliferation of CD4^+^ T cells. We have also observed similar bystander stimulatory effect of PM on phagocytosis of microbial components by macrophages (data not shown). Moreover, these findings are in an agreement with previous reports demonstrating the positive effect of PM on Ag endocytosis by DC, namely, DEP facilitated Ag internalization through the enhanced expression of scavenger receptors on DC (Miyata and van Eeden 2011; Taront et al. 2009).

Independently of the effect of PM on DC functions, we have demonstrated markedly enhanced immunogenicity of OVA treated with PM, the main finding in the present study. It was established by the significant enhancement of T cell proliferation associated with the increased production of IFN-γ, the major cytokine of Th1 cells (Lee et al. 2021). IFN-γ itself can efficiently up-regulate the class II antigen-presenting pathway and thus promote peptide-specific activation of CD4^+^ T cells (Giroux et al. 2003).

Hypothetically, the enhanced immunogenicity of PM-modified OVA might be achieved by the following mechanisms:(a) facilitation of OVA uptake (endocytosis) by APCs; (b) facilitation of OVA processing due to its increased susceptibility to proteolytic cleavage; c) more effective antigen presentation by enhancement of affinity of OVA 323–339 epitope to the specific TCR or by increased number of epitope–MHC complexes on the surface of an APC.

To explain the mechanisms of enhanced immunogenicity of PM-modified OVA, the following possibilities are discussed. Firstly, our results suggest that the increased resistance of PM-modified OVA to proteolytic digestion and increased thermal stability can exclude the role of OVA processing in its enhanced immunogenicity (Thai et al. 2004; Winter et al. 2020).

However, the alternative conclusion is also probable. Namely, the increased resistance of PM-modified OVA to the tested proteolytic enzymes was achieved at alkaline pH, while acidic pH occurs in late endosomes during antigen processing. Although our experimental conditions are distinct from those in vivo, the results suggest that PM-modified OVA, more resistant to proteolytic digestion, might be retained longer in APC cells after internalization. Consistent with this finding is the observation that DCs in vivo degrade internalized antigens slowly and thus keep non-degraded antigen for an extended period. Such limited lysosomal proteolysis also favors antigen presentation and makes the protein more immunogenic (Delamarre et al. 2005).

Moreover, the absence of cysteine and methionine, the primary targets for ROS, in the structure of OVA 323–339 epitope excludes the possibility of OVA (antigen) oxidation in this region. Importantly, amino acid 333 (alanine) was identified as the primary TCR contact residue for OT-II TCR-transgenic T cells, while alanine 331 was found to be an important secondary TCR contact residue (Robertson et al. 2000). Therefore, no epitope, but the carrier part of OVA protein seems to be the potential target for oxidative modification by PM (SRM 1648a or LAP).

Furthermore, the obtained results point to functional groups’ modification and conformational changes of the protein caused by exposure to PM as a reason of boosted immunogenicity. It is noteworthy that OVA treated with SRM 1648a elicited a stronger immune response compared to LAP, as evidenced by the increased cell proliferation and IFN-γ production. These results correlate with the identified protein modifications.

The question remains what structural changes of the carrier part of OVA protein are responsible for the enhanced immunogenicity of PM-modified OVA.

Metal ions extracted from the PM samples during dialysis may affect the microenvironment of OVA by increasing the surface tension and hydrophobicity (Zhang et al. 2019). Analysis of the concentration of sulfhydryl groups has shown that the exposure of OVA to PM led to reduced availability of free –SH groups compared to control protein treated with PBS only. The reduced number of thiol groups can be assigned to the generation of a single metal–sulfur bond or to cysteine residues oxidation. SRM 1648a is a multielement PM, which is able to liberate metal ions to an aqueous phase (Samek et al. 2017; Wądołek et al. 2021b). SRM 1648a is rich in active Cu(II) ions, but also in Hg(II) ions which can form nearly covalent bonds with thiols or Cd(II) ions. Such reactions potentially can be responsible for the decreased availability of thiol groups in ovalbumin (Bal et al. 2013; Oszajca et al. 2019; Wądołek et al. 2021a). Copper ions are known to diminish the number of –SH groups in two ways, either by forming a bridge with two cysteine residues resulting in a RS–Cu(II)–SR complex or playing a catalytic role in thiols oxidation. Observed increase in the number of carbonyl groups, slight reduction in the number of amino groups and additional oxidation of methionine residues (M212 and M274) may also point to metal-catalyzed oxidative protein modifications. Numerous reports support the notion that metal-catalyzed oxidation is the most important mechanism of protein oxidative damage and results in polypeptide backbone cleavage, cross-linking, and modification of amino acid side chains, all of which can result in a structural alteration and loss of protein functions (Cheignon et al. 2016; Maisonneuve et al. 2009). In the present study, we have shown that structural changes of OVA induced by dialysis against PM did not cause protein aggregation. On the contrary, the dialysis against PM resulted in a slight dimer dissociation, as evidenced by an increase in OVA monomeric to dimeric fractions ratio. Higher stability of OVA treated with PM was further confirmed in the trypsin digestion assay, which indicates resistance of PM-treated OVA to proteolytic cleavage. The observed stabilization of OVA might be explained by the binding of metal ions to the protein surface, which can increase protein stability due to the change in protein charge and induced electrostatic interactions (Ianeselli et al. 2010).

Although we have no direct evidence that the enhanced T cell response to PM-modified OVA was due to the increased uptake of modified OVA by APC, we have observed the increased protein level in DCs upon incubation with PM-modified OVA in cell culture medium containing 5% FBS (Fig. 6). Such culture condition (protein rich medium) was used to mimic the in vivo milieu of cells. These results point to the possibility that the protein uptake (endocytosis) by DCs might be facilitated in the presence of PM-modified OVA. Therefore, it is possible that the exposure to PM might result in the modification of serum proteins, which in turn might enhance/accelerate the overall endocytosis by APCs.

In conclusion, our major finding is the observation that PM-modified OVA demonstrates enhanced immunogenicity as evidenced by significantly higher response (proliferation) of OVA-specific T cells. To the best of our knowledge, it is the first report suggesting the adjuvant-like effect of air pollutants. Nevertheless, further research is needed to fully elucidate the mechanism of the observed phenomenon.

Finally, our findings suggest that inhaled airborne PM may not only lead to oxidative stress and the activation of systemic inflammation, but also may stimulate Ag-specific response to PM-modified serum proteins (Tan et al. 2011), especially to albumin (Khan 2022), the most abundant and major antioxidant serum protein. We have also demonstrated recently that inhaled PM accelerates and aggravates the symptoms of disease in the experimental model of rheumatoid arthritis (Nowak et al. 2022). It is possible that PM-dependent modification of other serum proteins may be responsible for the enhanced risk of autoimmune diseases in urban areas.

## Data Availability

The data that support the findings of this study are available from the authors upon reasonable request.
